# Genetic and functional studies of the *LMF1* gene in Thai patients with severe hypertriglyceridemia

**DOI:** 10.1016/j.ymgmr.2020.100576

**Published:** 2020-03-10

**Authors:** Wanee Plengpanich, Suwanna Muanpetch, Supannika Charoen, Arunrat Kiateprungvej, Weerapan Khovidhunkit

**Affiliations:** Endocrinology and Metabolism Unit, Department of Medicine and Hormonal and Metabolic Disorders Research Unit, Faculty of Medicine, Chulalongkorn University, Excellence Center in Diabetes, Hormone, and Metabolism, King Chulalongkorn Memorial Hospital, Thai Red Cross Society, Patumwan, Bangkok 10330, Thailand

**Keywords:** LMF1, Variants, Lipoprotein lipase, Triglyceride, Chylomicron, Chylomicronemia

## Abstract

Severe hypertriglyceridemia (HTG) due to chylomicronemia is associated with acute pancreatitis and is related to genetic disturbances in several proteins involved in triglyceride (TG) metabolism. Lipase maturation factor 1 (LMF1) is a protein essential for the maturation of lipoprotein lipase (LPL). In this study, we examined the genetic spectrum of the *LMF1* gene among subjects with severe HTG and investigated the functional significance of 6 genetic variants *in vitro*. All 11 exons of the *LMF1* gene were sequenced in 101 Thai subjects with severe HTG. For an *in vitro* study, we performed site-directed mutagenesis, transient expression in *cld* cells, and measured LPL protein and LPL activity. We identified 2 common variants [p.(Gly36Asp) and p.(Pro562Arg)] and 12 rare variants [p.(Thr143Met), p.(Asn249Ser), p.(Ala287Val), p.(Met346Val), p.(Thr395Ile), p.(Gly410Arg), p.(Asp433Asn), p.(Asp491Asn), p.(Asn501Tyr), p.(Ala504Val), p.(Arg523His), and p.(Leu563Arg)] in 29 patients. *In vitro* study of the p.(Gly36Asp), p.(Asn249Ser), p.(Ala287Val), p.(Asn501Tyr), p.(Pro562Arg) and p.(Leu563Arg) variants, however, revealed that both LPL mass and LPL activity in each of the transfected cells were not significantly different from those in the wild type *LMF1* transfected cells, suggesting that these variants might not play a significant role in severe HTG phenotype in our subjects.

## Highlights

• Among 101 subjects with severe hypertriglyceridemia (HTG), 2 common and 12 rare variants in the LMF1 gene were identified
• None of the 6 missense variants studied were associated with a reduction in lipoprotein mass or activity
• These rare variants in the *LMF1* gene may not play an important role in severe HTG phenotypes in the Thai population

## 1 Introduction

Severe hypertriglyceridemia (HTG) occurs when there is massive accumulation of triglyceride-rich lipoproteins, chylomicrons and/or very low-density lipoproteins, in the bloodstream. The presence of chylomicrons in fasting plasma samples results in chylomicronemia, which is associated with an increased risk of acute pancreatitis. The cause of chylomicronemia can be either monogenic or multifactorial [[1], [2], [3], [4]]. The monogenic form, called familial chylomicronemia syndrome (FCS), is a rare autosomal recessive disease due to the presence of bi-allelic pathogenic variants in several genes involved in triglyceride lipolysis. These candidate genes include *LPL*, *APOC2*, *APOA5*, *GPIHBP1* and *LMF1*, which encode, respectively, lipoprotein lipase, apolipoprotein C2, apolipoprotein A5, glycosylphosphatidylinositol-anchored high-density lipoprotein-binding protein 1, and lipase maturation factor 1 [2,5]. The multifactorial form of chylomicronemia, called multifactorial chylomicronemia (MCM) or multifactorial chylomicronemia syndrome (MCS), is an oligogenic or polygenic disorder due to heterozygous “smaller effect” variants in candidate genes along with predisposing environmental factors [[1], [2], [3], [4],^6^]. While FCS is associated with a high risk of acute pancreatitis, MCM or MCS is associated with a lower risk of acute pancreatitis but a higher risk of cardiovascular disease [4,7].

Lipase maturation factor 1 or LMF1 is a membrane-bound protein located in the endoplasmic reticulum and is essential for the posttranslation modification of LPL enzyme [8]. Bi-allelic loss-of-function mutations in the *LMF1* gene result in decreased LPL activity and FCS [[9], [10], [11]]. Several studies have reported the genetic variants in the *LMF1* gene in subjects with HTG, mainly in subjects of European descent [[9], [10], [11], [12], [13], [14], [15]].

Earlier studies identified homozygous nonsense variants in the *LMF1* gene, p.Tyr439Ter and p.Trp464Ter, causing premature termination codons and a profound reduction in LPL activity [9,10]. Surendran et al. later reported 8 missense variants in 15 patients with HTG (p.Gly36Asp, p.Arg230Glu, p.Arg264Cys, p.Arg351Glu, p.Arg354Trp, p.Arg364Gln, p.Arg523His, and p.Pro562Arg), but none of them was associated with a reduced LPL activity in *in vitro* experiments [12]. Recently, Serveaux Dancer et al. identified 19 nonsynonymous (18 missense variants and 1 nonsense variant, p.Trp464Ter) in the *LMF1* gene in 65 subjects among 385 patients with severe HTG. They examined the functionality change of 12 missense variants and showed that only 4 missense variants (p.Gly172Arg, p.Arg354Trp, p.Arg364Gln, and p.Arg537Trp) significantly reduced LPL activity in the culture media [14]. These reported *LMF1* variants were mainly discovered in populations of European descent with HTG.

In Asian populations, data on the *LMF1* variants in subjects with HTG are scarce. A small study in 26 Korean subjects with HTG reported 2 common variants (p.Gly36Asp and p.Pro562Arg) and 3 rare variants (p.Met346Val, p.Gly410Arg, and p.Gly541Arg) in the *LMF1* gene [15]. A larger study in 103 Chinese patients with severe HTG identified 8 rare variants in 11 subjects [16]. However, no functional data have been provided for these variants, therefore, it is still unclear whether they contribute to severe HTG in Asian populations.

In this study, we examined the genetic variants of the *LMF1* gene in 101 Thai subjects with severe HTG (fasting triglyceride levels >10 mM) using a resequencing approach and determined their functional significance *in vitro*.

## 2 Methods

### 2.1 Subjects

One hundred and one patients who had severe HTG, defined as fasting TG level ≥ 10 mM or 886 mg/dL on at least 2 occasions, and referred to the Endocrine Clinic at King Chulalongkorn Memorial Hospital, Bangkok, were included in the present study as previously described [17]. This group of subjects likely consisted of both FCS and MCS, so we did not exclude any subject who had secondary causes of HTG. Informed consent was obtained from each subject. The study was approved by the Ethics Committee of the Faculty of Medicine, Chulalongkorn University.

### 2.2 Laboratory determinations

EDTA plasma and serum samples were collected after 10–12 h of fasting. Lipid levels were measured using enzymatic methods in an automated system by Roche [17].

### 2.3 Genetic analysis

DNA was extracted by phenol-chloroform. Coding regions and intron-exon boundaries of the *LMF1* gene were amplified by PCR using primers shown in Supplement Table 1 and purified by ExoSap-IT (Amersham Biosciences). PCR products were sequenced using an ABI 3730XL DNA Analyzer (Applied Biosystems) at Macrogen (South Korea). The rs number of each variant was checked in the dbSNP 149 database (https://www-ncbinlm-nih-gov.gate2.inist.fr/snp/). The *Refseq* accession number for *LMF1* was NM_022773.4. The Human Genome Variation Society (HGVS) Recommendations for the description of sequence variants were followed [18]. The allele frequency of *LMF1* variants was examined using data from the Exome Aggregation Consortium (http://exac.broadinstitute.org) version 0.3.1 (all-population databases). Rare variants were defined as a minor allele frequency (MAF) <1% in the general population whereas common variants were those with a MAF ≥1%.

### 2.4 The *cld* mutant cell line

The *cld* mutant cell line used was a gift from Dr. M. Peterfy (University of California, Los Angeles, USA) [10]. The *cld* cells were maintained in DMEM-10% FBS. For the LPL assay, a total of 3 × 10^5^ cells were distributed in a 12-well plate (Falcon), each flat-bottomed well containing 0.8 ml DMEM-10% FBS and having a surface area of 3.8 cm^2^. Transfection was initiated 24 h after plating when cells reached ≥90% confluence.

### 2.5 Expression constructs and *LMF1* target sequences

The *LPL* expression construct, the secreted human placental alkaline-phosphatase (*SEAP*) reporter construct, the human wild-type *LMF1* construct and the mutant p.Tyr439Ter *LMF1* construct have been previously described [9]. In brief, the *LPL* cDNA was subcloned into the pcDNA6/V5-His expression vector (Invitrogen). The C terminus of the expressed LPL protein was fused with the V5 epitope tag as described [19]. *LMF1* sequences were subcloned into the pcDNA3.1 expression vector (Invitrogen). Therefore, the expressed LMF1 protein contained an N-terminal c-*myc* epitope tag. The following human *LMF1* target sequences were used: the wild type *LMF1* encoding the full-length 567 amino acid protein and the p.Tyr439Ter nonsense mutation, removing 127 amino acids from the C-terminus of LMF1. To normalize for transfection efficiency the *SEAP* reporter gene was subcloned into the pM1 expression vector (Roche).

We induced 6 missense variants [p.(Gly36Asp), p.(Asn249Ser), p.(Ala287Val), p.(Asn501Tyr), p.(Pro562Arg) and p.(Leu563Arg)] using mutagenesis primers (Supplement Table 2). The 3 variants [p.(Gly36Asp), p.(Pro562Arg), p.(Leu563Arg)] were most prevalent in Thai subjects in this study and the other 3 [p.(Asn249Ser), p.(Ala287Val), p.(Asn501Tyr)] were chosen since these was no previous information on the functional significance of these variants. The *LPL/SEAP* plasmid master mix was prepared and used throughout all experiments. All expression vector plasmids used in our study were prepared using the Miniprep kit (Qiagen) according to the manufacturer's instructions. Diluted plasmid solutions were quantitated using a NanoDrop 2000 spectrophotometer.

### 2.6 Cotransfection and cell harvesting

Each cotransfection experiment contained a mixture of *LPL* and *SEAP* constructs. Transfection of *cld* mutant cells was achieved using the Effectene® transfection reagent (Qiagen) at a DNA: reagent ratio of 1:10. Each well of a 12-well plate was transfected with 15 ng of the *LMF1* target sequence and a mixture of 0.8 μg and 0.2 μg of *LPL* and *SEAP*, respectively according to the manufacturer's instructions. At 24 h posttransfection, a sample of medium was taken for measurement of SEAP activity, and then heparin was added to a final concentration of 15 U/ml. At 48 h posttransfection, samples were removed for measurements of LPL mass and LPL activity. At the end of the experiment, cells were washed twice with PBS and lysed in a detergent-containing buffer (150 mM NaCl, 20 mM Tris-HCl, pH 7.5). After sonication and centrifugation, supernatants from the resulting lysates and medium samples were stored at −80 °C until use.

### 2.7 Detection and quantitation

SEAP activity was measured using the SEAP Reporter Assay kit (Roach). LPL mass and LPL activity were measured using the assay kit (Cell Biolabs). Secreted LPL activity was reported as mU/ml/h, normalized to SEAP activity.

### 2.8 Bioinformatic studies

Both the PolyPhen (http://genetics.bwh.harvard.edu/pph/: version 2.2.2) and Protein Analysis Through Evolutionary Relationships (PANTHER; www.pantherdb.org: version 13.1 released 2018-02-03) programs were used to determine dysfunction of the variants. PolyPhen determines the impact of nonsynonymous SNPs according to a position-specific independent counts (PSIC) score difference. The results denote three types, “probably damaging”, “possibly damaging” and “benign”, depending on risk. The PANTHER program estimates the amino acid changing (coding SNP) to cause a functional impact on the protein. It calculates the length of time (in millions of years or “my”) a given amino acid has been preserved in the lineage leading to the protein of interest. The longer the preservation time is, the greater the likelihood of functional impact is. The method is called PANTHER-PSEP (position-specific evolutionary preservation). The thresholds were: “probably damaging” (time > 450 my), “possibly damaging” (450 my >time > 200 my) and probably benign (time < 200 my) [20].

### 2.9 Statistical analysis

Data are presented as mean ± SD. One-way ANOVA with posthoc analyses was used to compare data among multiple groups. *P* value <.05 was considered statistically significant. Statistical analysis was performed using the SPSS software program (version 22, Chicago, IL).

## 3 Results

Clinical characteristics and lipid levels of 101 HTG subjects have been previously described [17]. The range of the triglyceride level was 892 to 10,840 mg/dL. The 3 most common secondary causes of HTG were diabetes mellitus (49%), alcohol use (36%) and HIV infection (22%). Thirteen subjects had history of acute pancreatitis. Rare variants in *LPL* or *APOA5* and/or the common *APOA5* p.(Gly185Cys) variant were found in 37% of this HTG group whereas no *APOC2* variant was identified [17]. In this study, we further identified 14 variants in the *LMF1* gene, all of which were missense variants, in 29 subjects. Two missense variants were common variants [p.(Gly36Asp) and p.(Pro562Arg)], whereas the other 12 were rare variants, including p.(Thr143Met), p.(Asn249Ser), p.(Ala287Val), p.(Met346Val), p.(Thr395Ile), p.(Gly410Arg), p.(Asp433Asn), p.(Asp491Asn), p.(Asn501Tyr), p.(Ala504Val), p.(Arg523His), and p.(Leu563Arg) as shown in Table 1. Among 29 subjects, 15 harbored only common variants, 5 harbored both common and rare variants, and the other 9 harbored only rare variants in the *LMF1* gene. There was no significant difference in the clinical characteristics among these 3 groups.

**Table 1:** Genetic variants identified in the *LMF1* gene in 101 patients with severe HTG.

rs number	Exon	Position	Variant	PolyPhen		PANTHER		Homozygous (n)	Heterozygous (n)	Allele frequency (ExAC browser)
				Prediction	Score	Prediction	Preservation time (millions of years)			
rs111980103	1	c.107G>A	p.Gly36Asp	Benign	0.000	Probably benign	1	1	4	1.82 × 10^−1^
rs375529211	2	c.428C>T	p.Thr143Met	Possibly damaging	0.773	Probably benign	1	–	1	1.89 × 10^−4^
rs766214202	6	c.746A>G	p.Asn249Ser	Probably damaging	0.966	Probably benign	1	–	1	1.95 × 10^−5^
rs755996576	6	c.860C>T	p.Ala287Val	Possibly damaging	0.637	Probably benign	1	–	1	5.41 × 10^−5^
rs201767825	7	c.1036A>G	p.Met346Val	Benign	0.001	Probably benign	1	–	1	2.63 × 10^−4^
rs186694298	8	c.1184C>T	p.Thr395Ile	Possibly damaging	0.591	Probably benign	1	–	2	4.01 × 10^−4^
rs199713950	8	c.1228G>A	p.Gly410Arg	Probably damaging	0.989	Probably benign	1	–	1	9.04 × 10^−4^
rs201927375	9	c.1297G>A	p.Asp433Asn	Benign	0.076	Probably benign	1	–	1	2.04 × 10^−4^
rs532127028	10	c.1471G>A	p.Asp491Asn	Possibly damaging	0.920	Probably benign	1	–	1	3.69 × 10^−4^
rs774296427	10	c.1501A>T	p.Asn501Tyr	Probably damaging	0.991	Probably benign	1	–	2	8.44 × 10^−6^
rs369478194	10	c.1511C>T	p.Ala504Val	Benign	0.110	Probably benign	1	–	1	1.70 × 10^−4^
rs151137164	11	c.1568G>A	p.Arg523His	Benign	0.359	Probably benign	1	–	1	2.96 × 10^−3^
rs4984948	11	c.1685C>G	p.Pro562Arg	Possibly damaging	0.754	Probably benign	176	–	17	5.11 × 10^−2^
rs199591347	11	c.1688T>G	p.Leu563Arg	Possibly damaging	0.500	Probably benign	1	–	3	4.24 × 10^−4^

All these known variants were all heterozygous, except for p.(Gly36Asp), which was heterozygous in 4 subjects and homozygous in 1 subject. Clinical characteristics of the subject with homozygous p.(Gly36Asp) variant were not different from those of heterozygous p.(Gly36Asp) variant. Among 29 subjects who harbored the *LMF* variants, 25 subjects (86%) already had documented common or rare variants in the *LPL* or *APOA5* gene which had been associated with HTG [17]. Considering only those who harbored rare variants in the *LMF1* gene (*n* = 14), 11 (78%) already had variants in the *LPL* or *APOA5* gene that could contribute to HTG.

**Fig. 1:**
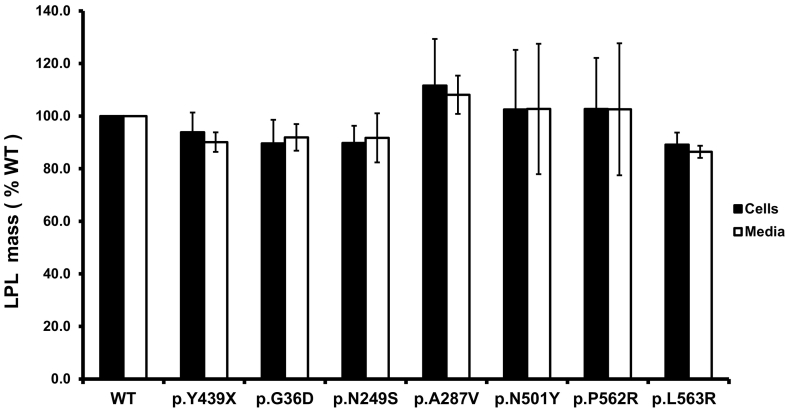
LPL mass in the cell lysate and media after expression of the p.(Gly36Asp), p.(Asn249Ser), p.(Ala287Val), p.(Asn501Tyr), p.(Pro562Arg) and p.(Leu563Arg) variants in *cld* cells. LPL mass (ng/ml) was measured in the cell lysates (black columns) and in the culture media (white columns) and expressed as percentage of wild type (WT). *N* = 3.

**Fig. 2:**
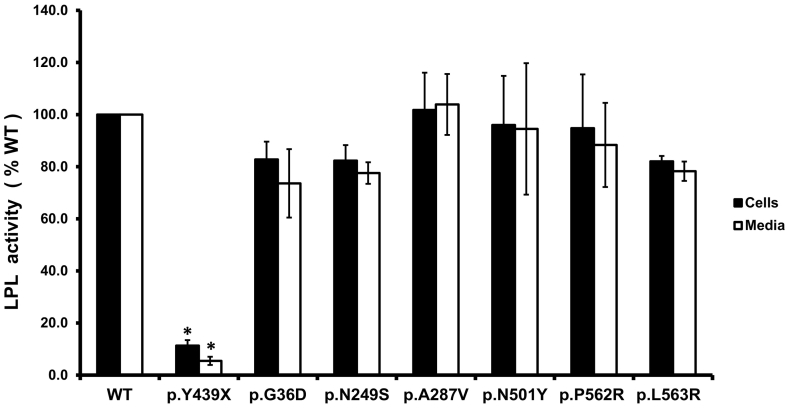
LPL activity in the cell lysate and media after expression of the p.(Gly36Asp), p.(Asn249Ser), p.(Ala287Val), p.(Asn501Tyr), p.(Pro562Arg) and p.(Leu563Arg) variants in *cld* cells. LPL activity (mU/ml/h) was measured in the cell lysates (black columns) and in the culture media (white columns) and expressed as percentage of wild type (WT). *: *P* < .05 *vs.* the wild-type *LMF1* DNA. N = 3.

We selected 6 variants, p.(Gly36Asp), p.(Asn249Ser), p.(Ala287Val), p.(Asn501Tyr), p.(Pro562Arg) and p.(Leu563Arg), to further characterize the functional significance by expression in *cld*-mutant hepatocytes in combination with an *LPL* expression vector. The characterized nonsense variant (p.Tyr439Ter) which had been shown to abolish LPL activity [9] was used as a positive control. As shown in Fig. 1, Fig. 2, the wild type *LMF1* rescued LPL activity in *cld* cells whereas the p.Tyr439Ter nonsense variant was unable to restore LPL activity even though LPL protein was present in abundance, suggesting the critical role of LMF1 in maturation of LPL as previously reported [9]. We then evaluated the LPL mass of these selected 6 variants and we found that there were no significant differences in the LPL mass between the wild type and these 6 variants (Fig. 1). We further examined the LPL activity and found that none of these *LMF1* variants led to a significant reduction in LPL activity in the cell lysates or the media (Fig. 2). One of the selected variants, p.(Pro562Arg), had previously been tested by others [12,14] and their results were similar to ours. Therefore, our *in vitro* study suggested that these selected 6 variants in the *LMF1* gene were not associated with a decrease in LPL function.

Unfortunately, no functional data are presently available for the other 5 missense variants [p.(Met346Val), p.(Thr395Ile), p.(Asp433Asn), p.(Asp491Asn), and p.(Ala504Val)]. However, among 6 subjects who harbored these 5 missense variants, 4 had a common c.-3A>G variant in the *APOA5* gene and 1 had a rare heterozygous p.(Leu279Val) variant in the *LPL* gene, both of which had been shown to contribute to HTG [17].

## 4 Discussion

In this study, we determine whether *LMF1* variants were associated with HTG in the Thai population. Our cohort of patients with severe HTG was a mixture of both FCS and MCM. We first sequenced the entire coding regions of the *LMF1* gene in 101 Thai patients and found 14 different variants. Two common missense variants, p.(Gly36Asp) and p.(Pro562Arg), were found along with 12 rare missense variants. Although some of these variants were predicted *in silico* to be detrimental to the function of the protein, our *in vitro* functional study showed that 6 of these variants were not pathogenic.

LMF1 is a protein located in the endoplasmic reticulum and is required for the maturation of LPL, thus loss-of-function variants in the *LMF1* gene are expected to affect LPL activity and predispose to chylomicronemia. A number of studies have identified several variants in the *LMF1* gene in subjects with HTG [[9], [10], [11], [12], [13], [14], [15]]. In those with FCS, some particular nonsense variants have been found to be causative, for example, p.Tyr439Ter, p.Tyr460Ter, and p.Trp464Ter [[9], [10], [11]]. In addition, a few other studies have reported several missense variants in subjects with varying degrees of HTG, most of which are from the populations of European descent [[12], [13], [14], [15], [16]].

Surendran et al. studied 85 subjects with HTG from the Netherlands and identified 8 common and rare missense variants in the *LMF1* gene, however, none of these variants were associated with a reduction in LPL activity *in vitro* [12]. Further studies from Canada and Spain also found a number of rare missense variants but no functional data were provided [13,21]. Recently, a study in 385 subjects from France reported 19 nonsynonymous variants and showed that only 4 of them were associated with a decrease in LPL activity [14].

Sequence variation in the human genome is race- and ethnic-specific. It is of note that all of the above studies have been performed in Caucasian populations [[9], [10], [11], [12], [13], [14],^21^]. In Asian populations, a small study from Korea identified 2 common and 3 rare variants in the *LMF1* gene [15]. A larger study in 103 Chinese patients with severe HTG recently reported 8 rare variants in 11 subjects [16]. Unfortunately, none of the above studies provided functional data, making it difficult to contribute these variants to the development of severe HTG or chylomicronemia.

Our study in 101 Thai subjects with severe HTG identified 2 common variants and 12 rare variants. Both of the 2 common variants [p.(Gly36Asp) and p.(Pro562Arg)] have been found in both the Caucasian and Asian populations [12,14,15]. We have also provided functional data for these 2 variants that they are not associated with a reduction in LPL activity (Fig. 2).

Regarding the rare variants, we did not detect any nonsense variants (p.Tyr439Ter, p.Tyr460Ter, and p.Trp464Ter) commonly reported among the Caucasian patients [[9], [10], [11]]. Instead, we have found 12 other missense variants. Three of these 12 missense variants [p.(Thr143Met), p.(Gly410Arg), and p.(Arg523His)] have already been found in Caucasian populations and *in vitro* studies have shown that they were not associated with a decrease in LPL activity [12,14]. We have provided functional data for the other 4 rare missense variants [p.(Asn249Ser), p.(Ala287Val), p.(Asn501Tyr), and p.(Leu563Arg)] that all of these 4 variants are not associated with a reduction in the secretion and/or activity of LPL. Unfortunately, no data are presently available for the other 5 missense variants we found in this study [p.(Met346Val), p.(Thr395Ile), p.(Asp433Asn), p.(Asp491Asn), and p.(Ala504Val)], and further experiments are required to examine the functionality of these variants. The p.(Asp433Asn) and p.(Asp491Asn) variants are close to p.Tyr439Ter and p.Trp464Ter, which have previously been associated with a profound reduction in LPL activity [9,10]. Similarly, the p.(Arg523His) variant is close to p.Arg537Trp, which has been associated with reduction in LPL activity [14].

At present, it should be noted that different *in vitro* systems exist to determine the functionality of *LMF1* variants and there may be differences between these models [9,12,14]. For example, p.Arg354Trp and p.Arg364Gln *LMF1* variants were shown to be detrimental to LPL activity in one study [14] but not in the other [12]. It is possible that different *in vitro* systems might have different detection capabilities and one system might be more sensitive over the others in detecting milder effects of certain *LMF1* variants. Certain *in vitro* systems may be able to detect variants with moderate effects, but not milder effects. Differences in cell culture systems (*cld* mutant hepatocytes *vs.* human embryonic kidney 293 T (HEK-293 T) cells), DNA quantity used for transfection, and incubation time might explain the discrepancies found between various studies.

Given that none of the variants in the *LMF1* gene that we studied are associated with a reduction in LPL activity and the majority of subjects who are carriers of the *LMF1* rare variants already have documented variants in either the *LPL* and/or *APOA5* genes which can contribute to HTG, we conclude that pathogenic variants in the *LMF1* gene do not play a major role in the development of severe HTG and the MCM phenotypes in our Thai subjects. Obviously, none of the LMF1 variants identified are causative for the FCS phenotype in some of our cohort. The result of our studies is in line with those from others in different populations [[12], [13], [14]] and also reinforces the importance in obtaining *in vitro* data to confirm the functional significance of the variants identified in patients.

There are some limitations of our study. First, we did not perform *in vitro* experiments of all *LMF1* variants identified and further experiments are definitely required to document their functional significance. Second, post-heparin LPL activity was not available in our study. Although post-heparin LPL activity is considered highly variable among assays and laboratories [6], it might be useful in documenting the *in vivo* functionality of the new variants identified. Third, our cohort of subjects are likely a mixture of FCS and MCM and the result might be different if only FCS patients were strictly enrolled.

In summary, we identified 2 common and 12 rare variants in the *LMF1* gene in 101 Thai patients with severe HTG, but an *in vitro* expression experiment showed that none of the 6 variants studied were pathogenic, suggesting that variants in the *LMF1* gene rarely contribute to severe HTG and chylomicronemia phenotypes in the Thai population.

## Declaration of Competing Interest

The authors report no conflicts of interest.

## Appendix A Supplementary data

Supplementary tables Supplement Table 1. Sequence of primers used to amplify exons and exon-intron boundaries of the *LMF1* gene. Supplement Table 2. Primers used for site-directed mutagenesis for the *LMF1* gene. Image 1
