# Supplementary material for: Genetic and functional studies of the LMF1 gene in Thai patients with severe hypertriglyceridemia
Source: Mol Genet Metab Rep. 2020 Mar 10;23:100576. doi: 10.1016/j.ymgmr.2020.100576 (PMC7068683; doi:10.1016/j.ymgmr.2020.100576)
Supplement: Supplementary file 1 — Supplementary tables Supplement Table 1. Sequence of primers used to amplify exons and exon-intron boundaries of the LMF1 gene. Supplement Table 2. Primers used for site-directed mutagenesis for the LMF1 gene. [file mmc1.docx]

Supplement Table 1. Sequence of primers used to amplify exons and exon-intron boundaries of the *LMF1* gene.

| Exon | Forward primer (5’to 3’) | Reverse primer (5’to 3’) |
| --- | --- | --- |
| 1 | GCTCAGGCTGGGAGAAAG | GTCGCCAGCAAGCCTTGT |
| 2 | TGCTGCAGGTTGGCTTTTCC | TGAAGATGGGTGGCTGCTGT |
| 3 | CGTGGGGAGGGGCTCCTGTT | CGCCCATCACTGCCCTCCGT |
| 4 | TCCTGCAGACCTGACCTTGC | AGAGGTGTGGTTCTGTCCCA |
| 5 | AAAGCAGCCCTGAATCTGCC | CAGAGCAAACCAGCTGCACT |
| 6 | TTGGCCCCTCTTAGCGTGGC | GGAAGGAGACCCTCAGCCTG |
| 7-8 | AGGCACCTCTGAGGCATCTG | ATGGGACCAGGACGAGGAGG |
| 9 | ATGAGACCCTGGGGCCACAG | TCCCACATGAGGCCCCTCCT |
| 10 | GGGCTACACGAAGCAGGCAG | GAGGTGGGCTATGAGGCAGG |
| 11 | GGTGCCGACTGGAACAGTTG | TGGGAGCCGCCACAGTATGT |

Supplement Table 2. Primers used for site-directed mutagenesis for the *LMF1* gene.

| Variants | Forward primer (5’to 3’) | Reverse primer (5’to 3’) |
| --- | --- | --- |
| p.(Gly36Asp) | GCCGGGGCGTGACCCCGCAGGCT | AGCCTGCGGGGTCACGCCCCGGC |
| p.(Asn249Ser) | GCCGATGCCCACTCCTGTGGCGT | ACGCCACAGGAGTGGGGCATCGGC |
| p.(Ala287Val) | CGGCCGGCGGGTGTGCATCATCC | GGATGATGCACACCCGCCGGCCG |
| p.(Asn501Tyr) | GCTGGCACACTACCCCTTCGCGG | CCGCGAAGGGGTAGTGTGCCAGC |
| p.(Pro562Arg) | CCGTGGGTGGCGTCTGCCCGGGC | GCCCGGGCAGACGCCACCCACGG |
| p.(Leu563Arg) | TGGGTGGCCTCGGCCCGGGCCCC | GGGGCCCGGGCCGAGGCCACCCA |
